# Validation of *Gastrochilus prionophyllus* (Vandeae, Orchidaceae), a new species from Yunnan Province, China

**DOI:** 10.3897/phytokeys.130.34555

**Published:** 2019-08-29

**Authors:** Xun-Feng Wu, De-Ping Ye, Bo Pan, Xiang-Qun Lin, Hong Jiang, Qiang Liu

**Affiliations:** 1 Yunnan Forestry Technological College, Jindian Road, Panlong District, Kunming, Yunnan 650000, China; 2 Southeast Asia Biodiversity Research Institute, Chinese Academy of Sciences, Yezin, Nay Pyi Taw 05282, Myanmar; 3 Forest Bureau of Pu’er, Yunnan, 665000, China; 4 Xishuangbanna Tropical Botanical Garden, Chinese Academy of Sciences, Mengla, Yunnan 666303, China; 5 Yunnan Academy of Forestry, Yunnan, 650000, China

**Keywords:** Orchidaceae, section *Microphyllae, Gastrochilus*, taxonomy, Yunnan

## Abstract

*Gastrochilus prionophyllus*, which was previously not validly published, is here validated. The species is described along with illustration and photos. Morphologically, the long and pendulous stem and distichous leaves of this new species indicate that it belongs to the sect. Microphyllae. It is unique in having thick fleshy leaves and margin significantly serrate, small flowers, reniform epichile and margin with dentations, thickened cushion on the central epichile and subconic hypochile. Meanwhile, a preliminary conservation status assessment according to IUCN Red List categories and criteria is given to the new species.

## Introduction

The genus *Gastrochilus* D. Don was established in 1825 (Epidenroideae; Vandeae; Aeridinea), which includes more than 62 species collectively distributed from India and Sri Lanka throughout Indochinese Peninsula, extending southwards to Indonesia and eastwards from China to Southern Japan (Chen et al. 2009; Kumar et al. 2014; Govaerts et al. 2016; Raskoti 2016; Averyanov et al. 2018; Liu et al. 2016, 2019; Liu and Gao 2018,). Some studies had speculated that *Gastrochilus* spread from tropical regions to temperate alpine regions (Fan et al. 2009; Liu et al. 2019). China is the diversity center of *Gastrochilus* and contains 42 species, of which 22 species are recognized as endemic to China, mainly in the Southern and the Southwestern parts of the country (Tsi 1996; Chen et al. 2009; Kumar et al. 2014; Liu et al. 2016, 2019; Liu and Gao 2018).

During our field investigation in the limestone forest of Southeast Yunnan Province, a species of *Gastrochilus* with serrate leaf was collected. After reviewing the relevant literature, two of the authors agreed the collection shares an identical morphology and provenance to the species presented and named previously as *G. prionophyllus* in The Wild Orchid in Yunnan (Xu et al. 2010). But it was never validly published, as neither provided a description nor cited the type specimen according to the *International Code of Nomenclature for algae, fungi and plants* (Shenzhen Code) (Turland et al. 2018). Here, the name of *Gastrochilus prionophyllus* is validly published. In addition, an illustration, a note on distribution, and affinities with other closely resembling species and IUCN conservation status are provided.

## Materials and method

Morphological observations of the new species were based on living plants (five individuals) and dried herbarium specimens (three specimens kept in the herbaria of HITBC) from two flowering seasons (2015 to 2016). All morphological characters were measured by using the vernier caliper. Both herbarium specimens and fresh material of *G. distichus* and *G. corymbosus* were examined. The conservation status of the new species was evaluated based on the guidelines of the International Union for Conservation of Nature (IUCN 2017).

## Taxonomic treatment

### Gastrochilus prionophyllus

Taxon classificationPlantaeAsparagalesOrchidaceae

H.Jiang, D.P.Ye & Q.Liu sp. nov.

154B5215BAD05E9591A23F4536B4EDDA

urn:lsid:ipni.org:names:60479348-2

**Figure 1.**
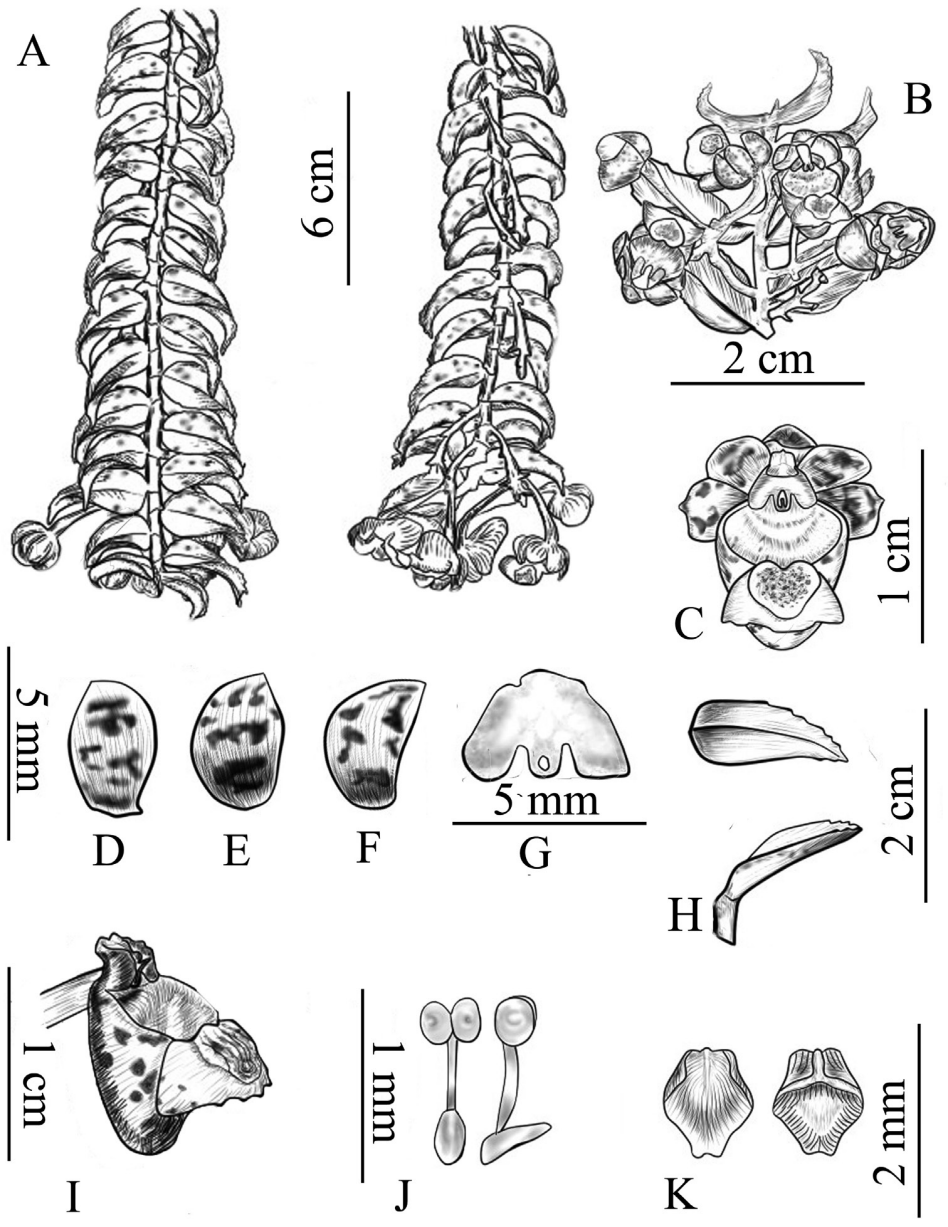
*Gastrochilus prionophyllus*. **A** Adaxial view and abaxial view of plant **B** inflorescence **C** front view of flower **D** lateral sepal **E** petal **F** dorsal sepal **G** front view of column **H** margin of leaf **I** lateral view of labellum and column **J** pollinarium **K** abaxial and adaxial anther cap. All from the type collection (Qiang Liu, *359*) and drawn by Bo Pan.

**Figure 2.**
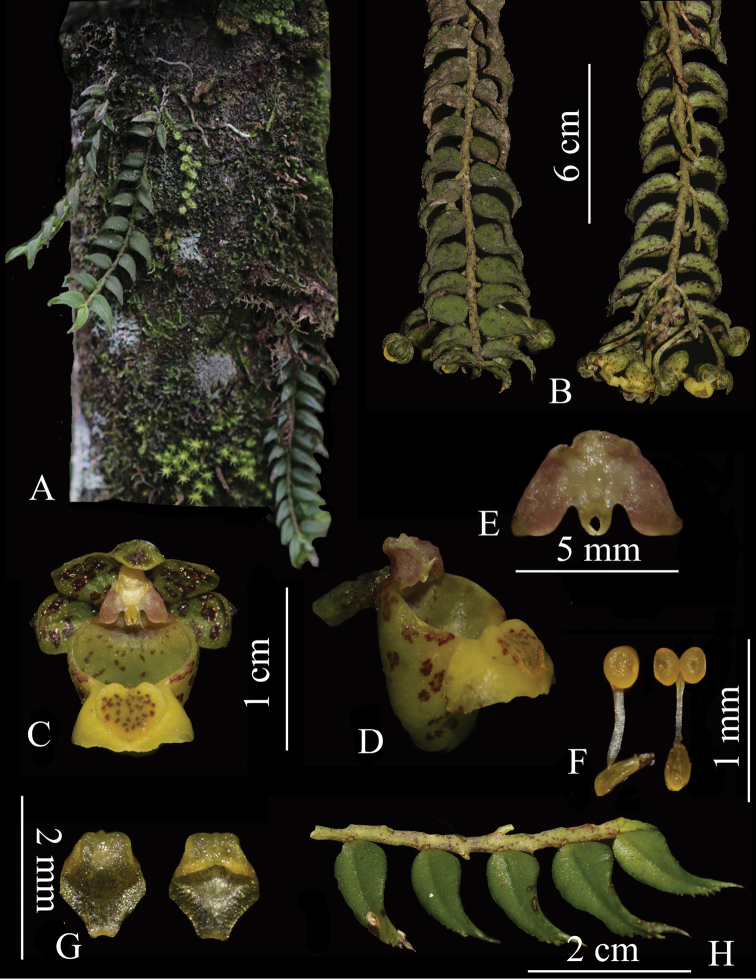
*Gastrochilus prionophyllus*. **A** Plant habit **B** plant **C** front view of flower **D** lateral view of labellum and column **E** front view of column **F** pollinarium **G** anther cap **H** variation of leaf margin (Photographed by Q. Liu).

**Figure 3.**
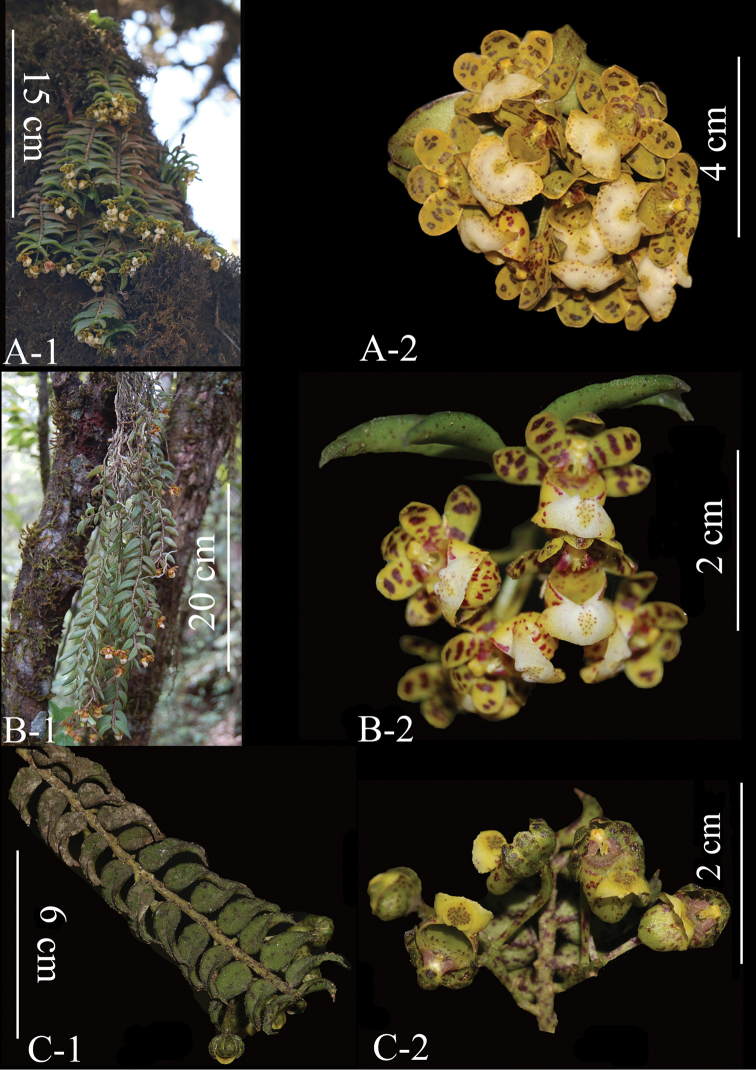
**A***Gastrochilus corymbosus* (**A-1** Plant **A-2** Inflorescence and flowers) **B***G. distichus* (**B-1** Plant **B-2** Inflorescence and flowers) **C***G. prionophyllus* (**C-1** Plant **C-2** Inflorescence and flowers) (Photographed by Q. Liu).

Figs 1 , 2 , 3C–3 , 1, C-2

Gastrochilus prionophyllys H. Jiang & D.P. Ye (2010: 475, Photos 694 a & b), *nom. inval.*

#### Diagnosis.

*Gastrochilus prionophyllus* is similar to *G. distichus* and *G. corymbosus*, but could be distinguished by having thick fleshy leaves and distinct serrate at the leaf margin, smaller flowers, reniform epichile with dentations at the margin and thickened cushion on the central epichile, and subconic hypochile.

#### Type.

CHINA. Yunnan Province: Malipo County, Xia jinchang town, limestone forest, 1550–1650 m a.s.l., epiphytic on tree trunks or on rocks, 15 Mar. 2016, Qiang Liu *359* (holotype, HITBC!)

#### Description.

Epiphytic herbs, stem pendulous, 10–15 cm long, ca. 1.25 mm in diameter, slender, unbranched with tiny red-purple spots. Leaves alternate, distichous, ovate, 0.9–1.3 × 0.3–0.5 cm, margin significantly serrate; leaf apex acuminate with 2 unequally awns; abaxial leaves with purple spots and sometimes on the adaxial leaves. Inflorescences several, opposite to leaves, subumbellate, 2–3-flowered; peduncle 0.9–1.1 cm, slender, upper part enlarged, lower part with 2 cupular sheaths; floral bracts ovate-lanceolate, 1.0–1.2 mm; pedicel and ovary 0.9–1.1 cm. Flower yellow-green, with reddish brown spots. Dorsal sepal concave, oblong-elliptic, 3.4–3.7 × 2.3–2.6 mm, apex obtuse; lateral sepal concave, oblong-elliptic, 3.6–4.2 × 2.6–3.0 mm, apex obtuse; petals subobovate, 3.5–4.1 × 2.3–2.5 mm, apex obtuse. Lip with an epichile and a saccate hypochile; epichile nearly reniform, 2.8–3.3 × 4.9–5.3 mm, adaxially glabrous, with a thicken central cushion and 2 conic calli near base, margin irregularly denticulate; hypochile subconic, laterally compresses, 5.0–5.7 mm tall, 4.5–5.0 mm in diameter, apex rounded. Column stout, ca. 2.5 mm long; anther cap narrowed into a beak toward apex; rostellum bilobed with acuminated tip, and arising a horn-like awn from the center of each lobe (obvious in the lateral view).

#### Etymology.

The specific epithet refers to the significantly serrate margin of leaf blades.

#### Distribution and habitat.

At present, the new species is only to be found in Malipo town, Yunnan, China. It was also recorded in Ha Giang province of North Vietnam according to Prof. L.V. Averyanov’s comment. *Gastrochilus prionophyllus* was found on the tree trunks at altitudes from 1550 to 1650 m in the limestone broad-leaved forests, which is dominated by *Quercus marlipoensis* Hu & W. C. Cheng (Fagaceae), *Q. utilis* Hu & W. C. Cheng (Fagaceae), *Platycarya strobilacea* Siebold & Zuccarini (Juglandaceae), *Manglietia grandis* Hu & W. C. Cheng (Magnoliaceae), *Eriobotrya japonica* (Thunberg) Lindley (Rosaceae) and *Podocarpus macrophyllus* (Thunberg) Sweet (Podocarpaceae), as well as an abundance of other orchid species, including *Eria coronaria* (Lindley) H. G. Reichenbach (Orchidaceae), *Paphiopedilum malipoense* S. C. Chen & Z. H. Tsi (Orchidaceae) and *Habenaria fordii* Rolfe (Orchidaceae).

#### Phenology.

*Gastrochilus prionophyllus* was observed in flowering from March to April in the wild.

#### Chinese name.

ju ye pen ju lan (锯叶盆距兰)

#### Additional specimens examined.

CHINA. Yunnan: Malipo County, Xia jinchang town, limestone forest, 1600m a.s.l., 4 Mar. 2016, Qiang Liu *214* (paratype, HITBC!); China. Yunnan. Malipo County, Babu village, limestone forest, 1650m a.s.l., 16 Jul. 2016, Qiang Liu *376* (HITBC!)

#### Conservation status.

At present, *Gastrochilus prionophyllus* is only known from two sites (Malipo County: Xia jinchang town and Babu village), around 50 individuals were discovered based on two years of botanical surveys. Although limestone forests have been seriously threatened by modern destructive human activities, not limited to quarrying and rubber plantation expansion (Sodhi and Brook 2006; Liu et al. 2015), the distribution of the new species lies in the landmine zone, a legacy from the Sino-Vietnamese War and the restricted zone keeps the habitat of limestone forest intact in Malipo county. Therefore, we expect that more individuals could be found in the adjacent forests extending along the China-Vietnam border. We suggest that the current conservation status of this new species is Data Deficient (IUCN 2017).

## Key to the species of Gastrochilus sect. Microphyllae

**Table:** 

1	Thick fleshy leaves and margin significantly serrate, epichile reniform and margin with dentation, and hypochile subconic	***G. prionophyllus***
–	Fleshy leaves and margin without serrate, epichile suborbicular or rhomboid and margin without dentation, and hypochile cupular	**2**
2	Epichile with thickened cushion on the center	**3**
–	Epichile without cushion on the center	**4**
3	Subumbellate inflorescence, orbicular cushion on the central epichile	***G. distichus***
–	Corymb inflorescence, diamond cushion on the central epichile	***G. corymbosus***
4	Hypochile narrower than epichile	**5**
–	Hypochile broader than epichile	**6**
5	Epichile with two ridges ranging from base to apex	***G. affinis***
–	Epichile membranous with longitudinal ridges centrally	***G. alatus***
6	Inflorescence 1 or 2-flowered; leaves without awns at apex	***G. fuscopunctatus***
–	Inflorescence 5 or 6-flowered; leaves with 1–3 short awns at apex	**7**
7	Epichile cordiform and without papillate	***G. kadooriei***
–	Epichile subobucular and with palillate	***G. pseudodistichus***

Note: The long and pendulous stem of this new species indicate that it belongs to the sect. Microphyllae (Tsi, 1996), and these species are easily confused by having similarly vegetative and floral characters, especially between *G. distichus* and *G. pseudodistichus*. Such as the specimens of *G. distichus*: QTT *73-896* (KUN!), ETM *4115* (KUN!) and Wang *63369* (AMES!) all were misidentified as the *G. pseudodistichus*. Although the new species is similar to *G. distichus* and *G. corymbosus* in vegetative and floral characters, it differs from *G. distichus* by having thick fleshy leaves with significant serrates margin, smaller flowers and sepals size less than 4 mm, and thickened cushion on the central epichile (vs fleshy leaves and margin entire, flowers large and sepals size more than 5 mm, and slightly thickened cushion on the central epichile in *G. distichus*) (Chen et al. 2009) (see Table 1 and Figure 3); differs from *G. corymbosus* by subumbellate inflorescence, reniform epichile and margin with dentations, orbicular central cushion on the epichile and subconic hypochile (vs corymb inflorescence, suborbicular epichile and margin entire, diamond-shaped cushion on the epichile and subcupular hypochile in *G. corymbosus*) (Das and Chanda 1988) (see Table 1 and Figure 3).

**Table 1.** Morphological comparison of *Gastrochilus prionophyllus* and its closely related species.

Character	*G. prionophyllus*	*G. corymbosus*	*G. distichus*
Habitat	Limestone forest	Subalpine rhododendron forest	Monsoon evergreen broad leaved forest
Plant length	10–15 cm	10–15 cm	10–30 cm
Leaf	Ovate, acuminate with 2 awns, margin serrate	Ovate, acuminate, without awn, margin entire	Lanceolate, acuminate with 2–3 awns, margin entire
Inflorescence	Subumbellate, peduncle 1.0 cm in length, 2–3 flowers	Corymb, peduncle1.3 cm in length, 4 flowers	Subumbellate, peduncle 2.5–3.0 cm in length, 2–4 flowers
Epichile	Reniform, with thickly and orbicular central cushion, and margin with dentations	Rhomboid, with diamond-shaped central cushion, and margin entire	Suborbicular, with orbicular central cushion, and margin entire
Hypochile	Subconic	Cupular	Subcupular
Flower period	March– April	October–November	January–May

## Supplementary Material

XML Treatment for Gastrochilus prionophyllus
